# Book Review: Vygotsky's Notebooks: A Selection

**DOI:** 10.3389/fpsyg.2020.595795

**Published:** 2021-02-16

**Authors:** Andrey Maidansky

**Affiliations:** ^1^Belgorod National Research University, Belgorod, Russia; ^2^Institute of Philosophy, Russian Academy of Sciences, Moscow, Russia

**Keywords:** cultural-historical psychology, instrumental method, semic method, higher mental functions, personality

Lev Vygotsky had a habit of writing down fresh thoughts on any sheets of paper that came into his hand. There remained hundreds of such notes and several notebooks from different years. Ekaterina Zavershneva and René van der Veer unscrambled, dated, and commented on these archival pieces. Each chapter of the book opens with a description of the texts collected in it and the related circumstances of Vygotsky's life.

In his notes, Vygotsky tries to get the hang of things for himself. That makes them particularly valuable. But we have only fragments of the movement of his thought. It is often extremely difficult to see the logic in them. More valuable is the help provided to the reader by the comments of Zavershneva and van der Veer. Another difficulty is that Vygotsky was an erudite in various fields of science and art. He has memory references, hidden quotes, and allusions at every turn. The commentators have managed to cope with almost all the difficulties of this kind.

In ch. 1–4 we find a young man reflecting on the historical fate of the Jewish people, on Jewish literature from Ecclesiastes to the beginning of the twentieth century, and on the image of Jew in world literature. Ch. 5 includes material for *The Psychology of Art*, which, for an uncertain reason, were not included in the book. Vygotsky examines various genres of writing and the way they provoke aesthetic reactions in the reader. Ch. 6 embraces Vygotsky's personal notes made during his trip to London at the 8th International Conference on the Education of the Deaf (July 1925).

Ch. 7–9 introduce the reader to Vygotsky's mental laboratory at the time when he was laying the foundations for the cultural-historical theory of higher psychological functions. The problem of method comes to the fore there. Vygotsky develops his “instrumental method” that includes the experimental technique of “double stimulation” (by an object and by a psychological tool) of memory, will, conceptual thinking and other functions.

The self, or “ego,” is defined as “the social in us.” “The ‘ego’ is formed after the model of the relationships between people” (p. 79). This statement becomes the cornerstone of cultural psychology.

Ch. 10 provides us with an opportunity to see how Vygotsky's idea of the systemic structure of the psyche arises. “The system is the basic concept of psychological analysis. Systems are the key to the person. In any case, the person does not consist of functions but of systems” (p. 141). He is particularly interested in the role of concepts in human mind as a psychological system. Vygotsky conceives the concept as an elemental “germ cell” of human freedom.

Several chapters are devoted entirely to the problems of child psychology: “The Anomalous Development of the Child” (ch. 11), “Observing Asya” (ch. 14), “Difficult Children” (ch. 27), “The Playing Child” (ch. 28).

Of particular interest are Vygotsky's notes from the internal conferences he organized. The theses of his own presentations and his notes on the reports of his collaborators demonstrate a lively thought at the moment of its birth and crystallization. We see hesitation and doubt, searching for arguments and reacting to criticism. All this is almost lost in publications but is highly important for our understanding his train of thought, the very process of creating a scientific theory.

In 1930, Vygotsky abruptly changed the course of his research. He moves on to a study of the systemic semantic structure of consciousness and the affective-emotional basis of psyche. His closest collaborator, Aleksei Leontyev, regards this as a departure from the Marxist stance in psychology; he insists on continuing the study of consciousness as a form of objective-practical activity. Leontyev left us his diaries and memoirs, and previously we only knew about that contest from his words. Now we have an opportunity to examine the situation through Vygotsky's eyes and to get to know how he answered Leontyev (ch. 15–17). In their mutual criticism and their subsequent “schism,” the fate of cultural-historical psychology was decided.

Ch. 18–21 contain research on the relationships between action, thought and speech. For this purpose, Vygotsky develops a “semic method.” He died before he had time to elucidate it in his published work. The only description of this method of analyzing the inner structure of sign operations can be found in his two notebooks. Vygotsky pays special attention to the phenomenon of schizophrenia. It is interpreted as the disintegration of action, word and thought that results in a “loss of inner freedom” (p. 315). He hoped it would provide a key to understanding the higher integrity of the free human personality.

In the last months of his life, Vygotsky studied a great deal of neuropsychology (ch. 26) and psychotherapy theory (ch. 29). His collaborator and co-author Alexander Luria would continue to develop the cultural-historical theory of localization of higher mental functions.

Overall, the book under review opens a new significant chapter in Vygotsky studies. His Notebooks allow and, I would even say, require us to take a new look at the history of cultural-historical psychology and provide highly valuable material for further development of this research program.

In terms of amount and quality of the reference apparatus, the book excels far and above any other English or Russian edition of Vygotsky's works. The editors of the volume, Ekaterina Zavershneva and René van der Veer, did a tremendous job. In conclusion, I can only express the hope that the work they started in the Vygotsky archive will continue at the same high level.

## Author Contributions

The author confirms being the sole contributor of this work and has approved it for publication.

## Conflict of Interest

The author declares that the research was conducted in the absence of any commercial or financial relationships that could be construed as a potential conflict of interest.

